# Molecularly imprinted polymer-modified carbon dots as a fluorescent probe for caffeic acid detection

**DOI:** 10.1039/d6ra03587f

**Published:** 2026-07-03

**Authors:** Yajuan Chang, Sunan Liu, Xiaojuan Jin, Ruien Shi, Yunsu Ma, Cheng Zhang, Xia Cheng, Ling Zhu, Xu Wang

**Affiliations:** a Department of Pharmacy, The Affiliated Suqian Hospital of Xuzhou Medical University Suqian Jiangsu 223800 China wangxu19910817@163.com; b Department of Pharmacy, The Affiliated Jiangyin Clinical College of Xuzhou Medical University Wuxi 214400 China zhuzhu09315@163.com; c Department of Pharmacy, Ruijin Hospital, Shanghai Jiao Tong University School of Medicine Shanghai 200000 China Chengxia199405@163.com; d Xuzhou Hospital of Traditional Chinese Medicine Affiliated to Nanjing University of Chinese Medicine Xuzhou Jiangsu 221000 China; e Xuzhou Medical University Xuzhou Jiangsu 221004 China

## Abstract

Caffeic acid is a phenolic acid compound widely present in nature. While beneficial to human health within a certain concentration range, excessive intake may lead to a series of health issues, such as liver and kidney function impairment. Therefore, developing a method for the precise and rapid detection of caffeic acid concentration in serum is of great significance for guiding rational clinical medication, drug monitoring, and daily health management. This study prepared a molecularly imprinted polymer (MIP) fluorescent probe for caffeic acid detection. Blue fluorescent carbon dots (bCDs) were synthesized *via* a one-step hydrothermal method using citric acid and diethylenetriamine as precursors. Subsequently, a molecularly imprinted silica shell was constructed on the bCDs' surface *via* a sol–gel method to obtain the bCDs@MIP fluorescent probe. Leveraging the specifically tailored recognition cavities created by molecular imprinting technology, the probe selectively captures caffeic acid, leading to fluorescence quenching. The degree of fluorescence intensity reduction exhibits a positive correlation with caffeic acid concentration, enabling its quantitative detection. The limit of detection (LOD) for caffeic acid was 0.15 µM. The probe was successfully applied to the determination of caffeic acid in human serum, providing a promising fluorescent strategy for caffeic acid analysis.

## Abstract 2

A molecularly imprinted fluorescent probe (bCDs@MIP) was developed for selective detection of caffeic acid *via* fluorescence quenching. The method shows a LOD of 0.15 µM and was successfully applied to human serum analysis.

## Graphical abstract

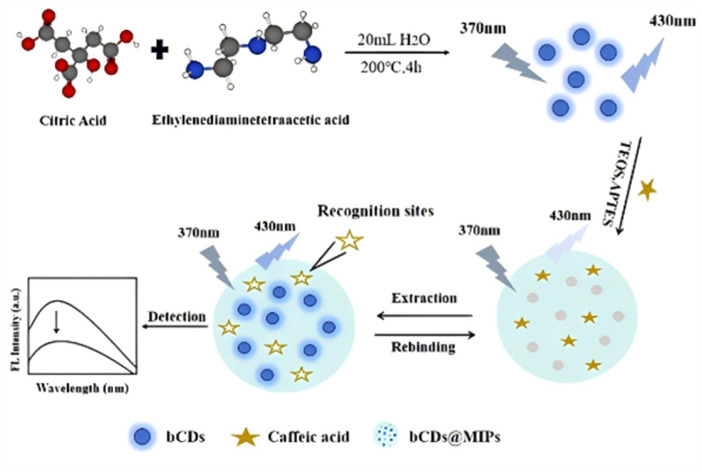

## 1. Introduction

Caffeic acid, a naturally occurring phenolic acid compound and a hydroxycinnamic acid derivative with the chemical name 3,4-dihydroxycinnamic acid, is widely found in plant-based foods such as fruits, vegetables, grains, and coffee. It possesses certain biological activities and potential health benefits, but its physiological effects exhibit significant dose dependency. Studies have confirmed that moderate intake of caffeic acid can significantly reduce the risk of type II diabetes^1^ and cardiovascular diseases^2^ through its antioxidant and anti-inflammatory properties. However, research reports indicate potential health risks associated with excessive intake. Caffeic acid may interfere with normal drug metabolism by inhibiting CYP450 enzyme activity,^3^ leading to hepatic lipid peroxidation damage and inducing apoptosis in renal tubular epithelial cells.^4^ This phenomenon has been linked to cases of liver injury induced by some herbal preparations,^5^ suggesting its potential toxicity may be dose-related. Furthermore, clinical observations have shown that serum caffeic acid levels are often significantly elevated in patients with metabolic syndrome, possibly reflecting abnormal regulation of polyphenol metabolism by gut microbiota.^6^ Therefore, accurate and rapid detection of caffeic acid concentration in human serum holds considerable scientific and practical value for guiding rational clinical drug use, studying its role in metabolic diseases, and even developing related early-warning biomarkers.

To date, various methods have been employed for the sensitive determination of caffeic acid, such as high-performance liquid chromatography (HPLC), gas chromatography-mass spectrometry (GC-MS), electrochemical techniques, and bienzymatic sensors. Although these analytical methods offer high sensitivity and accuracy, significant limitations remain when applied to complex biological matrices like human serum. For instance, HPLC requires complex pretreatment of serum samples,^7^ GC-MS often necessitates derivatization,^8^ and both methods rely on large instruments and specialized operation. Electrochemical methods are susceptible to interference from endogenous electroactive substances in serum (*e.g.*, ascorbic acid, uric acid), affecting the specificity for caffeic acid recognition.^9^ Bienzymatic sensors depend on the simultaneous immobilization and synergistic catalysis of two enzymes, whose activities are easily affected by environmental pH, temperature, and inhibitors in the sample matrix, resulting in limited long-term operational stability.^10^ Hence, developing a new method with simple pretreatment, strong anti-interference capability, and suitability for clinical analysis is of significant practical importance to meet the demand for rapid and highly selective detection of caffeic acid in serum samples.

Fluorescent sensing technology, owing to its advantages of high sensitivity, fast response, simple operation, and relatively simple instrumentation, shows great potential in the detection of biologically active small molecules. Among fluorescent materials, carbon dots (CDs), as a carbon-based nanomaterial, have been widely used for biomolecule detection due to their facile preparation, good optical properties, and low cost. The surfaces of CDs are typically rich in polar functional groups such as hydroxyl, carboxyl, and amino groups. These groups can engage in non-specific interactions with target analytes, such as hydrogen bonding, electrostatic interactions, and π–π stacking, through covalent or non-covalent modifications.^11^ For example, the caffeic acid molecule contains catechol hydroxyl groups and an acrylic acid side chain. Its hydroxyl and carboxyl groups can act as hydrogen bond donors or acceptors, while its benzene ring's π-electron system facilitates interactions with surface groups on CDs.^12^ Sun *et al.* synthesized CDs *via* a hydrothermal method using citric acid and urea as precursors. They established a caffeic acid detection method based on the fluorescence quenching effect of CDs, utilizing hydrogen bonding and π–π stacking interactions between the abundant polar groups on the CDs surface and caffeic acid molecules.^13^ Yan *et al.* constructed a fluorescent probe by encapsulating boron-doped carbon dots into a europium-based metal–organic framework (MOF). They achieved caffeic acid detection through fluorescence signal changes induced by coordination between boronic acid groups and the catechol hydroxyls of caffeic acid.^14^ However, these methods primarily rely on non-specific interactions between the target and the fluorescent material, making them susceptible to interference from structural analogs or coexisting substances in complex samples, which compromises selectivity and accuracy.

Molecular imprinting technology (MIT), a synthetic strategy mimicking antibody–antigen specific recognition, involves the formation of a polymer network around a template molecule using functional monomers and a cross-linker. Subsequent removal of the template leaves behind cavities in the polymer that are complementary in shape and functional group arrangement to the template, enabling highly selective recognition and enrichment of the target molecule.^15^ For instance, Tashakkori *et al.* prepared a magnetic molecularly imprinted polymer using caffeic acid as the template and a magnetic ionic liquid as the functional monomer for solid-phase extraction enrichment of caffeic acid, followed by quantitative determination using high-performance liquid chromatography-ultraviolet (HPLC-UV).^16^ Xing *et al.* used caffeic acid as the template, boronic acid-functionalized magnetic nanoparticles as the carrier, and tetraethoxysilane as the imprinting monomer to prepare surface-imprinted magnetic nanoparticles for specific recognition and enrichment of caffeic acid, combined with HPLC for quantitative analysis.^17^ While such methods combining molecularly imprinted materials with HPLC offer excellent selectivity and sensitivity, the subsequent HPLC quantification step is time-consuming, complex, and heavily reliant on equipment, making rapid detection difficult.

To address the insufficient selectivity of CDs and the cumbersome HPLC quantification step, researchers have combined MIT with CDs to construct molecularly imprinted fluorescent sensing platforms with both high sensitivity and selectivity. This strategy involves modifying a molecularly imprinted polymer (MIP) layer onto the CDs surface, effectively integrating the excellent fluorescence signal generation and transduction capabilities of CDs with the superior specific recognition ability of MIPs. It provides a promising solution for highly selective and sensitive fluorescent detection of targets in complex matrices. In recent years, the strategy of combining MIT with CDs has proven effective for detecting various analytes. For example, Hao *et al.* prepared CDs coated with a molecularly imprinted layer using 4-nitrophenol as the template. The specific recognition capability of the imprinted cavities significantly improved selectivity, achieving the limit of detection (LOD) as low as 0.06 µM.^18^ Liu *et al.* used the environmental estrogen bisphenol A as the template and coated CDs with a silica molecularly imprinted layer *via* a sol–gel method, constructing a fluorescent probe for detecting trace bisphenol A in environmental water and plastic products. This probe demonstrated far better selectivity than non-imprinted CDs, with a 1.5 nM LOD.^19^ These studies indicate that introducing MIT can effectively enhance the recognition capability and selectivity of CDs probes, offering new avenues for rapid and accurate detection of target analytes in complex systems.

Based on the above research background, this study synthesized a MIP fluorescent probe based on blue fluorescent carbon dots (bCDs). First, bCDs were prepared *via* a one-step hydrothermal method using citric acid and diethylenetriamine. Then, a silica MIP shell with specific recognition cavities was constructed on the bCDs surface *via* a sol–gel method using 3-aminopropyltriethoxysilane (APTES) as the functional monomer and tetraethoxysilane (TEOS) as the cross-linker. The resulting probe (bCDs@MIPs) selectively binds caffeic acid, leading to fluorescence quenching. This sensor undergoes methodological validation for stability, selectivity, and practicality and is applied to determine caffeic acid in human serum.

## 2. Experimental methods

### 2.1 Instruments

Fourier transform infrared (FT-IR) spectra and ultraviolet visible (UV-vis) spectra were measured using an IR affinity spectrophotometer and UV-2600 spectrophotometer, both from Shimadzu (Japan). Fluorescence spectra were collected using an F4600 fluorescence spectrophotometer (Hitachi, Japan), with both excitation and emission slit widths set at 5 nm. Transmission electron microscopy (TEM) images were obtained using an FEI-TECNAI G2 microscope operating at 200 kV. The fluorescence lifetime decay curve was measured using a steady-state/transient fluorescence spectrometer (Edinburgh FLS1000).

### 2.2 Reagents

Ammonia solution (H_5_NO, 25–28%) was purchased from Shanghai Ron Reagent Co., Ltd. l-Histidine (His, 99%) was from Shanghai Sigma-Aldrich Trading Co., Ltd. Diethylenetriamine (C_4_H_13_N_3_, 97%), citric acid monohydrate (C_6_H_8_O_7_·H_2_O, 99.5%) and chlorogenic acid (C_16_H_18_O_9_, 95%) were from Shanghai Aladdin Biochemical Technology Co., Ltd. Absolute ethanol (C_2_H_5_OH, analytical grade), acetone (C_3_H_6_O, analytical grade), methanol (CH_4_O, analytical grade), anhydrous calcium chloride (CaCl_2_, analytical grade), and magnesium chloride hexahydrate (MgCl_2_·6H_2_O, analytical grade) were from Sinopharm Chemical Reagent Co., Ltd. Caffeic acid (C_9_H_8_O_4_, 98%), APTES (C_9_H_23_NO_3_Si, 99%), TEOS (C_9_H_20_O_4_Si, 98%), glacial acetic acid (C_2_H_4_O_2_, 99.8%), sodium ion standard solution (Na^+^, 1000 µg mL^−1^), potassium chloride (KCl, 99.8%), d-(+)-glucose anhydrous (glucose, 99.5%), gallic acid (99%), ferulic acid (C_10_H_10_O_4_, 99%) and coumaric acid (C_6_H_4_O_4_, 97%) were from Shanghai Macklin Biochemical Co., Ltd. All aqueous solutions were prepared using Mill-Q ultrapure water (18 MΩ cm).

### 2.3 Preparation of bCDs

bCDs were synthesized according to a reported method^20^*via* a one-pot hydrothermal synthesis. In a 30 mL stainless steel autoclave with a Teflon liner, 1.2 g of citric acid monohydrate and 600 µL of diethylenetriamine were dissolved in 20 mL water. The autoclave was heated at 200 °C for 6 h. After cooling, the supernatant was collected by centrifuging at 8000 rpm for 10 min. Then, ethanol and acetone (volume ratio 1 : 3) were added for washing. The mixture was centrifuged at 8000 rpm for 10 min, and the precipitate was collected. The washing step was repeated three times. Finally, the precipitate was dried in a vacuum oven at 50 °C for 48 h to obtain bCDs.

### 2.4 Preparation of bCDs@MIPs fluorescent probe

bCDs@MIPs were synthesized according to a reported method^21^*via* a sol–gel process on the bCDs surface. First, 100 mg caffeic acid, 400 µL APTES, and 30 mL ethanol were sonicated and stirred under N_2_ for 0.5 h in a 100 mL three-necked flask. Then, 4 mg of the bCDs and 990 µL of TEOS were added. After mixing, 200 µL of ammonia solution mixed with 800 µL of water was added to initiate the polymerization reaction, which continued for 24 h. After the reaction, the product was centrifuged at 10 000 rpm for 10 min to collect the precipitate. The template caffeic acid was removed by multiple cycles of ultrasonication and centrifugation using methanol/glacial acetic acid (90 : 10, v/v) as the eluent, until no caffeic acid could be detected in the washing solvent by UV-Vis spectrophotometry. Finally, the obtained solid was dried in a vacuum oven at 60 °C for 12 h, yielding white bCDs@MIPs powder. Non-imprinted polymer-coated carbon dots (bCDs@NIPs) were prepared identically without caffeic acid.

### 2.5 Caffeic acid determination using bCDs@MIPs probe

First, a bCDs@MIPs solution with a concentration of 1.5 mg mL^−1^ was prepared using ultrapure water as the solvent and ultrasonicated before use to ensure uniform dispersion. Then, caffeic acid standard solutions (0, 1, 2, 20, 100, 200, 400, 1000, 1600 µM) were prepared using absolute ethanol. 200 µL of the bCDs@MIPs solution was mixed with 200 µL of caffeic acid standard solution, resulting in final caffeic acid concentrations of 0, 0.5, 1, 10, 50, 100, 200, 500, and 800 µM. The mixtures were vortexed and then reacted on a shaker for 15 min. After the reaction, the fluorescence intensity of each sample was measured at 430 nm with an excitation wavelength of 370 nm. A standard curve was plotted using the ratio of the fluorescence intensity of the blank sample (*I*_0_) to that of the sample containing caffeic acid (*I*), *i.e.*, *I*_0_/*I*, against the caffeic acid concentration.

### 2.6 Real sample detection

Serum caffeic acid was determined *via* standard addition. Human serum was diluted 10-fold with water. 100 µL caffeic acid solutions were added to 100 µL diluted serum, giving final concentrations of 0, 0.8, 1.6, 2.4 µM. The serum samples spiked with standard solutions were then processed according to the procedure in Section 2.5 to determine the caffeic acid content. The spike recovery rate, after subtracting the endogenous baseline, is calculated according to the following formula: recovery (%) = [(*C*_found, spiked serum_ − *C*_found, unspiked serum_)/*C*_spiked_] × 100%, which reduces the influence of the serum matrix and endogenous caffeic acid on the measurement results.

## 3. Results and discussion

### 3.1 Principle of the bCDs@MIPs fluorescent probe

**Scheme 1:**
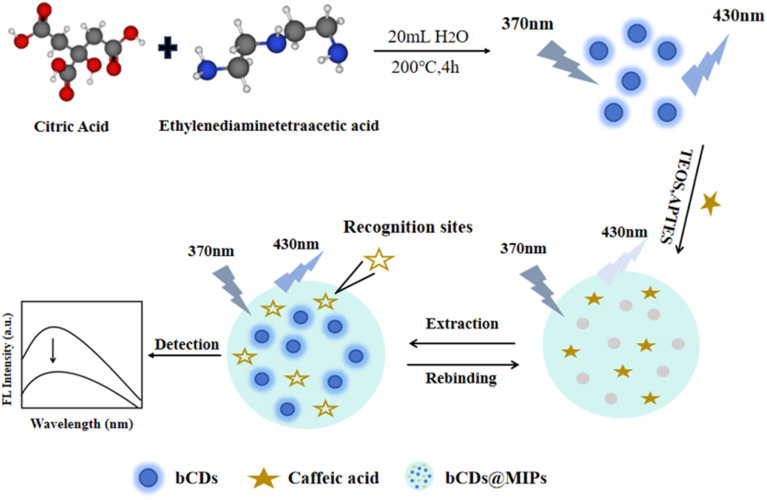
Schematic diagram of caffeic acid detection using bCDs@MIPs.

This study constructed a bCDs@MIPs fluorescent probe for caffeic acid detection and successfully applied it to detect caffeic acid in human serum. As shown in Scheme 1: first, bCDs were prepared *via* a one-step hydrothermal synthesis. The prepared bCDs exhibited a maximum fluorescence emission peak at 430 nm. Subsequently, using caffeic acid as the template molecule, it interacted with the functional monomer APTES *via* hydrogen bonding and van der Waals forces to form a “template-monomer” complex. Through a sol–gel reaction with the cross-linker TEOS, a silica network was formed, encapsulating the template molecule on the bCDs surface. After template removal, imprinted cavities complementary to caffeic acid in spatial structure and functional group distribution were left within the MIPs layer, yielding bCDs@MIPs with specific recognition sites for caffeic acid. Upon addition of caffeic acid, the fluorescence of bCDs@MIPs was quenched. The degree of fluorescence quenching was positively correlated with the caffeic acid content in the system. Therefore, quantitative detection of caffeic acid could be achieved by monitoring the change in fluorescence intensity of bCDs@MIPs.

### 3.2 Material characterization

**Fig. 1:**
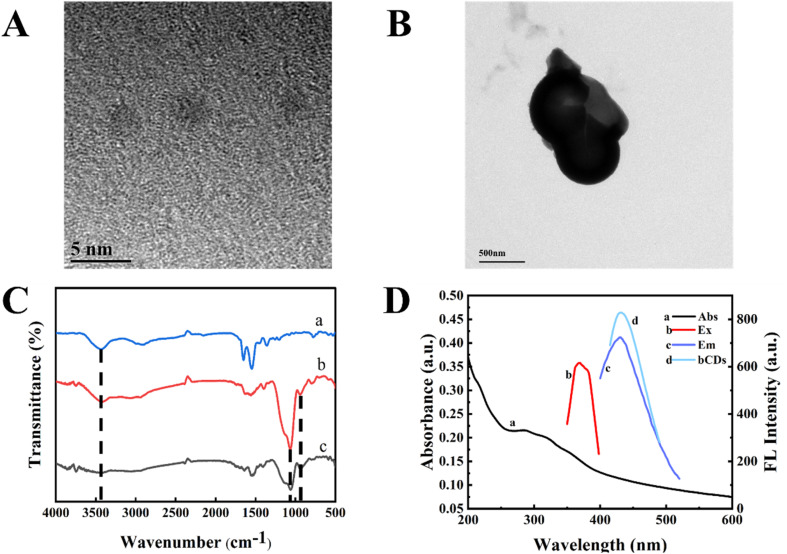
(A) and (B) TEM images of bCDs and bCDs@MIPs, respectively. (C) FT-IR spectra: curves a–c correspond to bCDs, bCDs@MIPs, and bCDs@NIPs, respectively. (D) Curves a–d correspond to the UV-Vis absorption spectrum, fluorescence excitation spectrum, fluorescence emission spectrum of bCDs@MIPs, and the fluorescence emission spectrum of bCDs, respectively.

To verify the successful synthesis of bCDs and bCDs@MIPs and investigate their morphological features, TEM characterization was performed first. The results, shown in Fig. 1A and B, reveal that bCDs are spherical and uniformly dispersed with a particle size of approximately 3 nm, while bCDs@MIPs are spherical with a particle size of about 500 nm. Further structural and optical property characterizations were conducted. Fig. 1C shows the FT-IR spectra of bCDs, bCDs@MIPs, and bCDs@NIPs. Curve a for bCDs exhibit a broad and strong absorption band around 3400 cm^−1^, corresponding to the stretching vibrations of surface hydroxyl (–OH) and amino (–NH_2_/–NH–) groups. These polar groups provide crucial binding sites for subsequent molecular imprinting, stabilizing the template molecule *via* hydrogen bonding or electrostatic interactions, laying the structural foundation for constructing the imprinted cavities.^22^ Curve b and c are the FT-IR spectra of bCDs@MIPs and bCDs@NIPs, respectively. Their peak positions are essentially identical. A strong broad peak at 1100–1000 cm^−1^ corresponds to Si–O–Si asymmetric stretching. A small peak near 792 cm^−1^ corresponds to Si–O–Si symmetric stretching, confirming siloxane formation. Furthermore, compared to bCDs, the O–H/N–H stretching vibration peaks in the imprinted materials are significantly weakened, likely due to the coverage of bCDs surface hydroxyl/amino groups by the silica shell. These results confirm the successful modification of the molecularly imprinted polymer on the bCDs surface. Fig. 1D presents the UV-Vis absorption spectrum and fluorescence properties of bCDs@MIPs. Curve a shows characteristic absorption at 250–350 nm, corresponding to the π–π* transition of the aromatic π-system in bCDs. Curves b–d show fluorescence spectra. Under 370 nm excitation, bCDs@MIPs emit at 430 nm. This emission is consistent with bCDs pre-imprinting, indicating the process did not significantly alter fluorescence.

### 3.3 Feasibility of the experiment

**Fig. 2:**
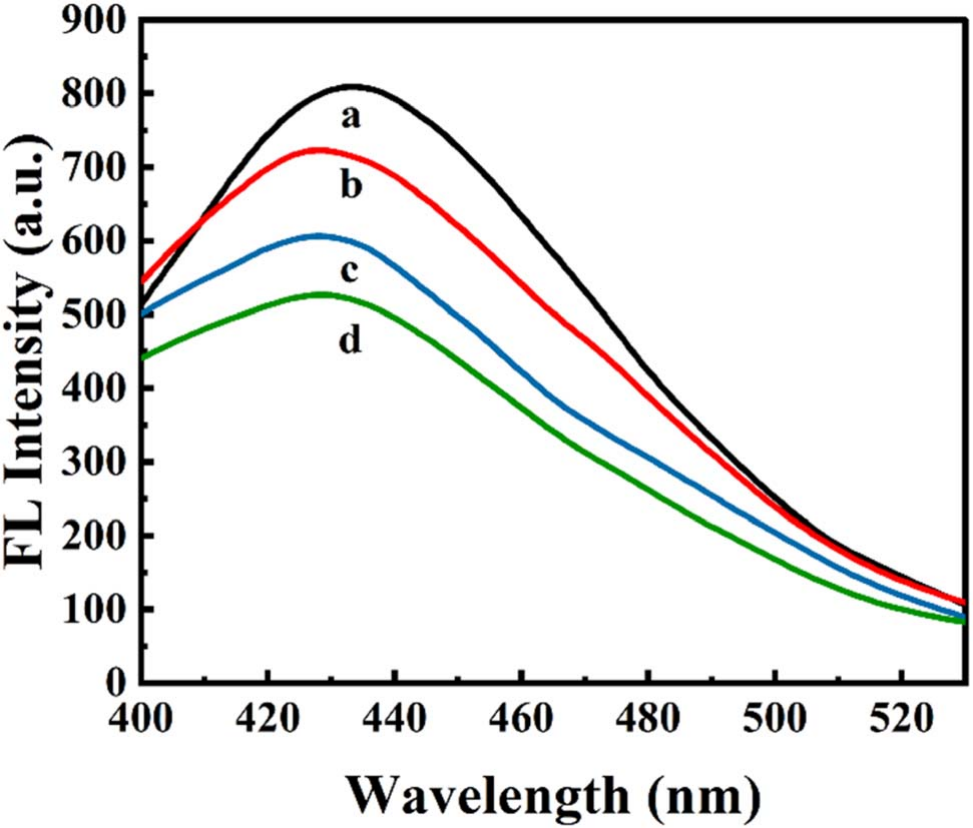
(a) Fluorescence spectrum of bCDs@NIPs; (b) and (d) fluorescence spectra of bCDs@MIPs after and before template elution, respectively; (c) fluorescence spectrum of bCDs@MIPs after adding 200 µM caffeic acid.

Feasibility was confirmed by fluorescence detection of bCDs@MIPs under different conditions and bCDs@NIPs. As shown in Fig. 2, bCDs@NIPs (curve a) had strong fluorescence. With unremoved template, bCDs@MIPs (curve d) showed significantly reduced fluorescence due to occupied sites hindering charge transfer. After template elution, bCDs@MIPs (curve b) fluorescence recovered near bCDs@NIPs level, indicating successful cavity construction. When 200 µM caffeic acid is added to the system, the fluorescence of bCDs@MIPs (curve c) is quenched again. This is attributed to the specific binding of caffeic acid to the imprinted cavities, triggering a charge transfer process where bCDs@MIPs act as an electron acceptor and caffeic acid acts as an electron donor. The reproducible “quench–recover–re-quench” pattern indicates: bCDs@MIPs accurately construct specific sites *via* MIT. These sites specifically bind caffeic acid, inducing a detectable fluorescence response. And the charge transfer mechanism is stable. This supports subsequent detection applications, proving feasibility.

### 3.4 Fluorescence lifetime analysis and quenching mechanism

**Fig. 3:**
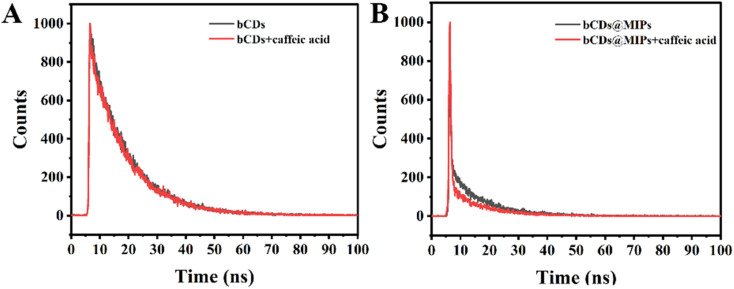
(A) Time-resolved fluorescence lifetime decay curves of bCDs and bCDs after the addition of caffeic acid; (B) time-resolved fluorescence lifetime decay curves of bCDs@MIPs and bCDs@MIPs after the addition of caffeic acid.

To further investigate the fluorescence quenching mechanism, we measured the time-resolved fluorescence lifetime decay curves of bCDs and bCDs@MIPs before and after the addition of caffeic acid. As shown in Fig. 3, after the addition of caffeic acid, the average fluorescence lifetime remains almost unchanged, indicating that there is almost no interaction between free bCDs and caffeic acid. In contrast, for bCDs@MIPs, the average fluorescence lifetime decreased from 10.37 ns to 8.86 ns after the addition of caffeic acid, suggesting that the binding of caffeic acid to the imprinted cavities promotes the non-radiative decay process of the excited state.^22,23^ The shortening of the fluorescence lifetime indicates that dynamic quenching is involved in this quenching process. Meanwhile, after caffeic acid is specifically recognized by the imprinted cavities, a ground-state complex of bCDs@MIPs–caffeic acid may be formed, leading to static quenching. Therefore, the fluorescence quenching of caffeic acid by bCDs@MIPs can be attributed to a mixed quenching mechanism involving both static and dynamic quenching.

### 3.5 Condition optimization

#### 3.5.1 Optimization of bCDs@MIPs preparation

**Fig. 4:**
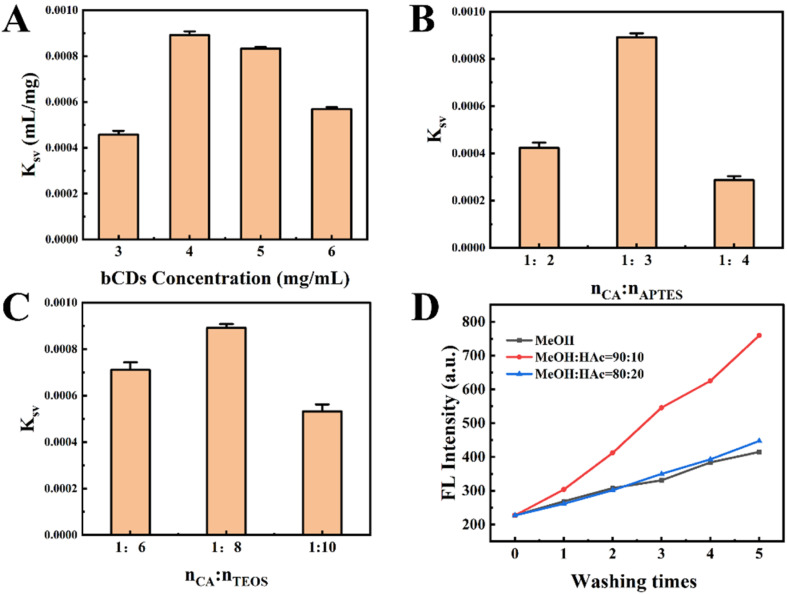
(A) Optimization results for bCDs amount (bCDs concentrations: 3, 4, 5, 6 mg mL^−1^). (B) Optimization results for functional monomer ratio (*n*_CA_ : *n*_APTES_: 1 : 2, 1 : 3, 1 : 4). (C) Optimization results for cross-linker ratio (*n*_CA_ : *n*_TEOS_: 1 : 6, 1 : 8, 1 : 10). (D) Optimization results for elution solvent (eluent : MeOH; MeOH : HAc = 90 : 10; MeOH : HAc = 80 : 20).

Preparation conditions were optimized using *K*_sv_ as an index. First, the bCDs amount was optimized. As shown in Fig. 4A, *K*_sv_ initially increased and then decreased with bCDs concentration from 3 to 6 mg mL^−1^, peaking at 4 mg mL^−1^. Therefore, 4 mg mL^−1^ was selected as the optimal bCDs amount. Next, the molar ratio of the functional monomer APTES to the template caffeic acid (CA) (*n*_CA_ : *n*_APTES_) was optimized. Ratios 1 : 2, 1 : 3, 1 : 4 were compared (Fig. 4B). *K*_sv_ was highest at 1 : 3, selected as optimal. Subsequently, the molar ratio of the cross-linker TEOS to the template CA (*n*_CA_ : *n*_TEOS_) was examined. As shown in Fig. 4C, *K*_sv_ peaked at 1 : 8, selected as optimal. Finally, the template elution conditions were optimized (Fig. 4D). Methanol : acetic acid (90 : 10) gave highest fluorescence recovery after same washes, effectively removing template. Therefore, methanol : acetic acid = 90 : 10 was selected as the optimal elution solvent.

#### 3.5.2 Optimization of bCDs@MIPs detection

**Fig. 5:**
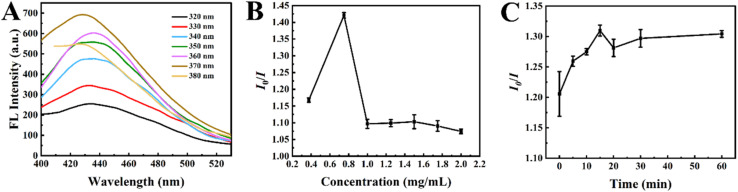
(A) Optimization results for excitation wavelength (excitation wavelengths: 320, 330, 340, 350, 360, 370, 380 nm). (B) Optimization results for bCDs@MIPs amount (bCDs@MIPs concentrations: 0.375, 0.75, 1.00, 1.25, 1.50, 1.75, 2.00 mg mL^−1^). (C) Optimization results for reaction time (reaction times: 0, 5, 10, 15, 20, 30, 60 min).

Detection conditions were optimized. As shown in Fig. 5A, fluorescence intensity gradually increased with increasing excitation wavelength, reached a maximum at 370 nm, and then decreased. Therefore, 370 nm was selected as the optimal excitation wavelength. The bCDs@MIPs probe concentration was then investigated (Fig. 5B). The *I*_0_/*I* was tested after adding 200 µM caffeic acid to different bCDs@MIPs concentrations. *I*_0_/*I* rose significantly from 0.375 to 0.75 mg mL^−1^, then fell rapidly. It fluctuated but trended downward from 1–2 mg mL^−1^. Therefore, 0.75 mg mL^−1^ was determined as the optimal probe concentration. Finally, the reaction time was optimized (Fig. 5C). *I*_0_/*I* gradually increased with increasing reaction time, reached a maximum at 15 min, and then began to decrease and gradually stabilized with further increases. Thus, 15 min was chosen as the optimal reaction time.

### 3.6 Methodological validation for caffeic acid detection

**Fig. 6:**
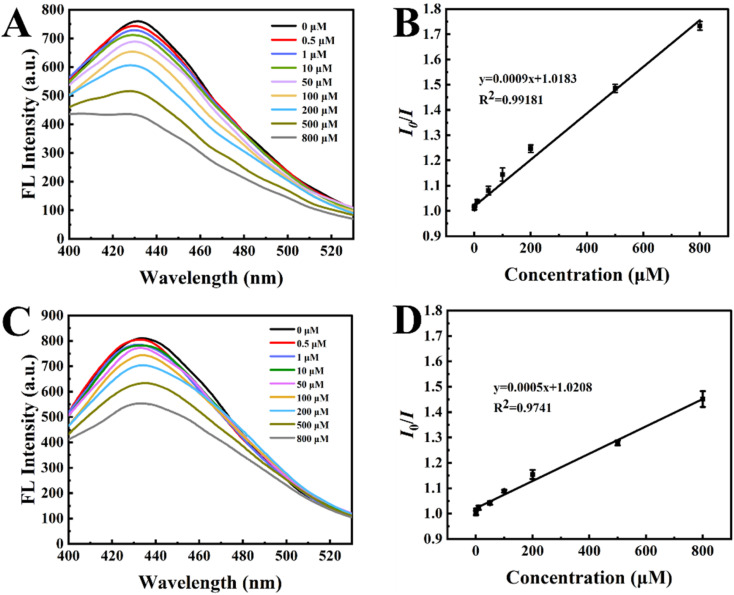
(A) Fluorescence spectra of bCDs@MIPs with different concentrations of caffeic acid. (B) Linear plot of the fluorescence intensity ratio *I*_0_/*I* of bCDs@MIPs *versus* caffeic acid concentration. (C) Fluorescence spectra of bCDs@NIPs with different concentrations of caffeic acid. (D) Linear plot of the fluorescence intensity ratio *I*_0_/*I* of bCDs@NIPs *versus* caffeic acid concentration.

Under optimal conditions, the prepared bCDs@MIPs fluorescent probe was used for the quantitative analysis of caffeic acid. As shown in Fig. 6A, the fluorescence intensity of bCDs@MIPs at 430 nm gradually decreased with increasing caffeic acid concentration, showing a negative correlation. Fig. 6B shows that the fluorescence intensity ratio *I*_0_/*I* had good linearity with concentration from 0.5–800 µM. The linear equation was *y* = 0.0009*x* + 1.0183 (*R*^2^ = 0.99181). The LOD, calculated as 3*σ*/*k* (where *σ* is the standard deviation of the blank sample, *n* = 10, and *k* is the slope of the calibration curve), was 0.15 µM. To evaluate the imprinting effect, the responses of bCDs@MIPs and bCDs@NIPs to caffeic acid were compared. As shown in Fig. 6C and D, although both exhibited fluorescence quenching with increasing caffeic acid concentration, the quenching degree of bCDs@MIPs was significantly higher than that of bCDs@NIPs, indicating that the specific recognition cavities formed during the imprinting process effectively enhanced the probe's selective recognition ability for caffeic acid. To assess the sensitivity of this fluorescent probe for caffeic acid quantitative detection, its LOD was compared with reported methods. As shown in Table 1, the LOD obtained in this study is comparable or superior, indicating that the prepared molecularly imprinted fluorescent probe offers good detection sensitivity for caffeic acid.

**Table 1:** Comparison of the probe performance of this work with those previously reported method

Analytical method	LOD (µM)	Linear range (µM)	Reference
Electrochemistry (FeCo@NPC/GCE)	0.14	0.4–110	24
Fluorescence (GQDs/G-quadruplex/hemin)	0.2	2–350	25
Electrochemistry (CuZnO_*x*_/MWCNTs/GCE)	0.155	1–100	26
Electrochemistry (F-GO/GCE)	0.018	0.5–100	27
Electrochemistry (Cu,Ni-BTC/GCE)	0.70	2–25	28
Fluorometric assay with bCDs@MIPs	0.15	0.5–800	This work

Furthermore, the precision and accuracy of the quantitative analysis method for caffeic acid were evaluated. Based on the 0.5–800 µM linear range, low, medium, high concentrations were 1.0, 20.0, 700.0 µM. Intra-day and inter-day accuracy and RSD were calculated (Table 2). For all concentrations, accuracy ranged 98.6–103.9%; RSD ranged 1.4–9.9%. This met the requirements for biological sample detection. These results indicate that the developed fluorescent probe can be used to determine caffeic acid content in real samples.

**Table 2:** Precision and accuracy of caffeic acid determination

Analyte name	Nominal concentration (µM)	Intra-run (*n* = 3)			Inter-run (*n* = 9)		
		Found (µM)	RSD (%)	Accuracy (%)	Found (µM)	RSD (%)	Accuracy (%)
Caffeic acid	1.0	0.99	8.3	99.0	1.02	5.8	102.2
	20.0	20.70	9.9	103.5	20.78	8.7	103.9
	700.0	692.92	1.4	98.9	699.83	2.2	98.6

**Fig. 7:**
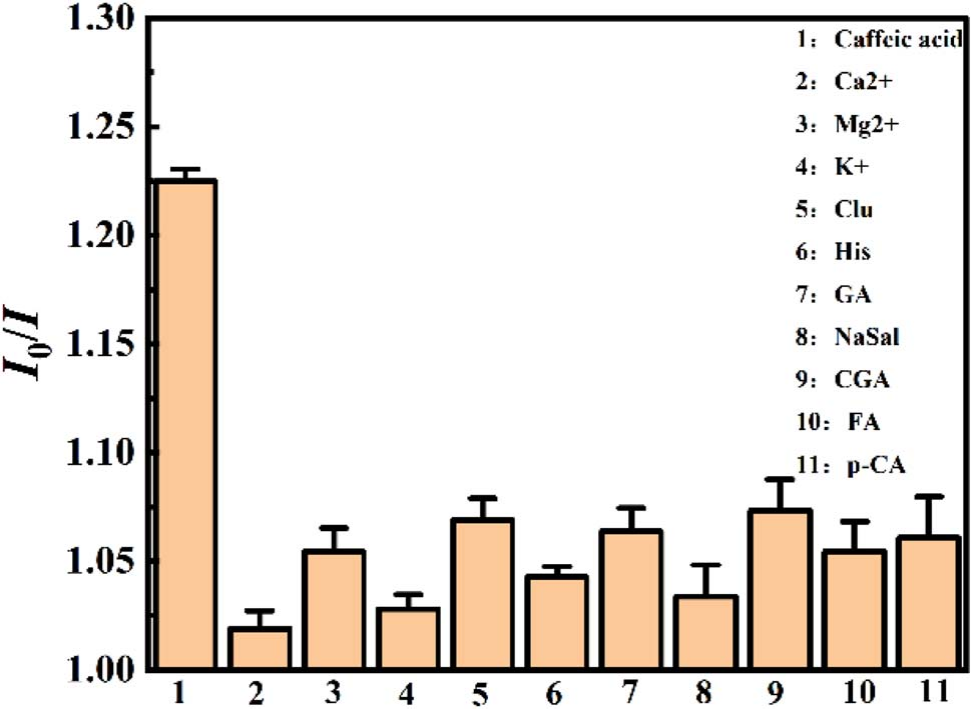
Selectivity of the fluorescent probe. Final concentrations: Ca^2+^, Mg^2+^, K^+^, Glu, His, GA, NaSal, CGA, FA and p-CA all 1 mM.

Selectivity was verified against interferents: Ca^2+^, Mg^2+^, K^+^, glucose (Glu), histidine (His), gallic acid (GA), sodium salicylate (NaSal), chlorogenic acid (CGA), ferulic acid (FA) and coumaric acid (p-CA). The results were compared with the response to caffeic acid. As shown in Fig. 7, the bCDs@MIPs fluorescent probe exhibited a significant response to caffeic acid but almost negligible responses to other interferents, indicating high selectivity of the bCDs@MIPs probe for caffeic acid.

### 3.7 Determination of caffeic acid in human serum

This experiment aimed to detect caffeic acid content in human serum. Considering the complex matrix effects, the standard addition method was chosen to eliminate matrix interference. As shown in Table 3, the spiked recoveries for caffeic acid in serum ranged from 98.3% to 105.0%, with RSDs ranging from 1.8% to 5.6%. These results indicate that the fluorescent probe possesses good sensitivity and can be used for detecting caffeic acid in human serum.

**Table 3:** Determination of caffeic acid content in human serum samples (*n* = 3)

Sample	Spiked (µM)	Found (µM)	Recovery (%)	RSD (%)
Serum	0.00	0.81	—	2.1
	0.80	1.65	105.0	5.6
	1.60	2.42	100.1	2.6
	2.40	3.17	98.3	1.8

## 4. Discussion

In summary, this study constructed a bCDs@MIPs fluorescent probe using caffeic acid as template, APTES as monomer, TEOS as crosslinker *via* sol–gel. The probe's *I*_0_/*I* ratio linearly related to caffeic acid concentration, with 0.15 µM LOD. Methodological validation results demonstrated that the probe possesses high sensitivity and selectivity for caffeic acid determination and is suitable for real sample detection. Moreover, the probe was successfully applied to detect caffeic acid in human serum. These results indicate that the constructed probe holds good application prospects for the rapid and selective detection of caffeic acid in complex biological samples.

## Ethical statement

All experiments were performed in accordance with the Declaration of Helsinki. This study, which involved human serum samples provided by Xuzhou Cancer Hospital, was approved by the Ethics Committee of Xuzhou Cancer Hospital (Approval No.: 2022-02-056-K01). Written informed consent was obtained from all participants.

## Conflicts of interest

The authors declare that they have no conflicts of interest.

## Data Availability

All data supporting this study are provided in full in this paper.
